# Research on Residual Strength and Evaluation Methods of Metal Aircraft Stiffened Panel Structures with Perforations

**DOI:** 10.3390/ma19071441

**Published:** 2026-04-03

**Authors:** Antai Ren, Tao An, Teng Zhang, Yitao Wang, Liying Ma

**Affiliations:** Aviation Engineering School, Air Force Engineering University, Xi’an 710043, China

**Keywords:** battle damage, tensile fracture tests, aluminum alloy, stiffened panels, ductile damage, residual strength evaluation, net section failure criterion

## Abstract

**Highlights:**

- Analyze and partially derive the fracture mechanisms of stiffened panels and the interaction mechanism between skin and stiffener.
- Investigate the effects of damage location (flange, web, or skin) and damage size on the residual strength of stiffened panel structures.
- Propose modifications to the residual strength evaluation method based on the net-section failure criterion, reducing a 3D problem to a 2D one.

**Abstract:**

This study investigates the interaction between the skin and stiffeners under tension and the structural failure mechanisms of aluminum alloy stiffened panels after battle damage, employing an integrated approach of experimental testing and numerical simulation. The variation in the residual strength of the stiffened panels with the characteristics of ruptures was explored, and an assessment method for residual strength was proposed based on the net-section failure criterion. The results indicate that the residual strength of the stiffened panels is closely related to the location and size of the rupture. For panels with ruptures of equal area, the residual strength is lowest for those with web damage, followed by those with flange damage, and highest for those with skin damage only. By employing an area-based conversion method, the three-dimensional stiffened panel was simplified to a two-dimensional plate. A stress averaging coefficient was introduced for large eccentric ruptures, while a conversion factor was applied for small eccentric ruptures to modify the residual strength assessment. The results demonstrate high accuracy. This study provides an efficient and precise method for evaluating the residual strength of damaged stiffened panels, offering a theoretical basis for aircraft battle damage repair.

## 1. Introduction

During combat, aircraft are often subjected to damage inflicted by various lethal weapons [1]. Missiles containing numerous fragments in their warheads detonate when approaching the aircraft, releasing explosion shock waves and high-velocity fragments to damage the aircraft structure. Since the intensity of explosion shock waves attenuates rapidly with distance [2], impact damage from high-velocity fragments constitutes the primary damage sustained by aircraft in combat [3]. High-velocity fragments mainly penetrate and damage targets that account for most of the aircraft surface. The aircraft surface, composed of aircraft panels, forms a large number of penetration holes under fragment impact. Such panel holes usually need rapid repair after the aircraft returns to base to enable its redeployment. Aircraft repair is closely associated with the evaluation of the residual strength of damaged structures or the strength of repaired structures. Therefore, structural strength assessment and damage repair of aircraft panels with penetration holes are important factors for improving aircraft combat effectiveness and maintaining the integrity of the aircraft formation [4,5].

To enhance the combat effectiveness of aircraft, maintain the integrity of aircraft formations, and improve the redeployment capability of damaged aircraft, repairs shall be carried out on the damaged structures of aircraft. Based on the post-repair structural strength, battle damage repair of aircraft typically employs equal-strength repair. However, given the limited resources and urgent time constraints in wartime scenarios, if the aircraft requires immediate deployment for subsequent missions, sub-strength repair or even non-strength repair (with plans for future repairs when time permits) may be adopted, provided the structural strength meets the requirements of the next mission [6]; nevertheless, decisive conclusions regarding whether the aircraft can be redeployed after simple repair necessitate relatively accurate battle damage assessment of the structure. Non-strength simple repair reduces stress concentration at the edges of perforations or cracks by temporarily grinding or cutting torn edges and hole-boundary cracks, resulting in smooth circular holes post-repair. Consequently, assessing the residual strength of smooth circular perforation damage on aircraft structures holds significant importance for reducing temporary repair time during wartime operations.

After redeployment, aircraft with damage simply repaired into smooth circular holes may be required to frequently perform high-intensity maneuvers (such as missile evasion). Some key load-bearing components on the aircraft (e.g., aircraft skin or fuselage panels) need to sustain large instantaneous loads. The aircraft structure with circular hole damage after simple repair may suffer from static strength failure due to instantaneous high tensile stress. Meanwhile, time is critical for aircraft during wartime, and the repair of damaged structures must be completed within 24 h. This forces aircraft that cannot be fully repaired to be redeployed with damage (only simple and temporary treatment can be applied to the damaged structures). The time limitation in wartime and the constraints on aircraft operational intensity allow an appropriate reduction in the strength requirements for damaged structures. As long as the residual strength of the aircraft structure meets the criteria, the damaged aircraft is permitted to perform the next short-term temporary mission. Moreover, fatigue damage has a negligible effect on the residual strength of damaged aircraft operating for a relatively short period. Considering the above two conditions, the residual strength evaluation of aircraft structures after combat damage is dominated by static strength [7].

Based on the above discussions regarding aircraft combat damage emergency repair, the residual strength evaluation of aircraft panel structures with smooth circular holes is of great significance for guiding combat damage emergency repair and aircraft redeployment. At present, the primary methods for residual strength evaluation of aircraft after combat damage are the analytical method and the finite element method. Given the urgent time constraints of wartime combat damage assessment, the analytical method usually employs a conservative yet simple and rapid evaluation scheme. Due to time constraints in wartime damage evaluation, analytical methods often adopt conservative yet simplified approaches. The traditional net-section yield criterion [8] allows conservative and rapid assessment of fracture in plastic material sections with smooth circular holes by comparing the average net-section stress (perpendicular to the load direction) against the material’s yield strength to determine failure. To further exploit structural utilization, the United States modified this criterion by replacing yield strength with ultimate tensile strength. However, this method has low evaluation accuracy and can hardly meet the requirements for accurate and effective evaluation of more complex structures. Wu et al. [9] derived three methods for evaluating the residual strength of metal panels with smooth circular holes based on linear elastic theory, elastoplasticity, and the net-section yield criterion, introducing stress concentration factors and residual strength coefficients. Case studies revealed that method selection depends on relative damage length and material fracture toughness: elastoplastic theory and the net-section yield criterion suit large-damage scenarios in ductile materials, while linear elastic theory applies to brittle materials. However, some data required by this method are difficult to obtain, which limits its universality. Ren et al. [10] adopted a combined approach of experiments and finite element simulation to conduct an in-depth study on the influence of hole parameters on the residual strength of flat plates. They introduced a stress averaging coefficient to modify the net-section failure criterion, and the derived analytical formula can accurately evaluate the residual strength of metallic flat plates with holes and be partially extended to single-stiffened panels. Nevertheless, this method is only applicable to flat plate structures with holes, and it does not further investigate the variation law and evaluation method of the residual strength of stiffened panels with holes. The above analytical methods can evaluate the structural residual strength through formula calculation, but they still suffer from low accuracy of evaluation results, complicated evaluation procedures, and difficulties in dealing with relatively complex structures. To address the problems of the analytical method and with the rise of the finite element method, finite element analysis has gradually become the dominant approach in combat damage assessment owing to its convenient and efficient computation. Li et al. [11] studied failure modes and loads of 2XXX/7XXXX-series ductile aluminum alloys under static tension, proposing a static strength criterion for perforated metal structures under local high stress. Their Abaqus models showed that the hole reduction coefficient is primarily determined by the difference between yield and ultimate stress—larger differences yield smaller coefficients. Wang et al. [12] combined ballistic tests of circular, square, and rod fragments penetrating panel structures with LS-Dyna simulations. Using restart techniques, they applied tensile loads to fragment-damaged panels until failure, elucidating the mechanism by which fragment damage degrades residual strength. The Finite Element Method (FEM) uses finite element software Abaqus 2024 to simulate and analyze the residual strength of structures. Callinan et al. [13] conducted a study on the residual strength of carbon fiber reinforced composites under ballistic impact. Ballistic tests were carried out using projectiles of various calibers. Non-destructive testing revealed that the damage range was dominated by projectile diameter, while the influence of impact velocity was negligible. By modifying the characteristic length model combined with finite element analysis, the stress redistribution mechanism induced by delamination was revealed, providing support for military aircraft structural design and battle damage repair. Zhang et al. [14] took 7075-T6 aluminum alloy thin sheet, the mainstream structural material of unmanned aerial vehicles, as the research object. Using high-velocity penetration tests, quasi-static tensile tests, fracture microanalysis and finite element simulation, they revealed the damage formation mechanism of high-velocity fragment penetration and clarified its degradation law on structural residual strength. A modified fracture mechanics evaluation method was proposed, and an explicit-quasi-static coupled simulation model was established to accurately reproduce the whole process of penetration damage and tensile failure. However, the finite element method also has inherent shortcomings in evaluating structural residual strength. When using finite element software to simulate and predict structural residual strength, a large number of finite element models need to be established and their accuracy verified, which consumes considerable preliminary preparation time and is unfavorable to the timeliness of combat damage emergency repair.

Among the above methods, the analytical method lacks simple and effective evaluation expressions for complex structures, while the finite element simulation method is rather time-consuming. At present, studies on the influence law of the location and size of penetration holes on the residual strength (failure load) of panels are still insufficient, and simple and effective evaluation expressions for the residual strength assessment of complex panel structures are also lacking. This study investigates the influence of perforation location and size on the static fracture load of stiffened panels. It designs three types of aluminum alloy double-stringer panel specimens and six types of triple-stringer panel specimens with varying perforation locations and sizes for quasi-static tensile fracture tests. The study analyzes the effects of flange and web perforations on the fracture failure modes and residual strength variation patterns of stiffened panels. Using finite element analysis (FEA) with a ductile damage criterion to simulate tensile fracture, the research accurately evaluates the maximum fracture load of pre-perforated specimens and extends the experimental conditions. Based on strain nephograms and FEA simulations, the force transmission paths, interaction modes, and failure patterns during panel fracture are analyzed in detail. Finally, a modified residual strength assessment method based on the net-section failure criterion is proposed. This paper fills the research gap on the residual strength law of stiffened panel structures with penetration holes at different locations and sizes, provides a theoretical basis and evaluation method for the residual strength assessment of post-combat damage structures, and improves the capability of aircraft combat damage emergency repair.

## 2. Materials and Methods

This study designs three types of aluminum alloy double-stiffener panels and six types of aluminum alloy triple-stiffener panels with varying pre-fabricated rupture hole locations and sizes for quasi-static tensile fracture tests, aiming to investigate the maximum tensile fracture load and the variation patterns of the load–displacement curves for stiffened panels containing pre-fabricated ruptures.

The panel is made of 2A12-T4 aluminum alloy with high strength and toughness, the stiffeners of ultra-high strength and brittle 7A04-T6 aluminum alloy, and the rivets of lightweight and high-strength 2A10-T4 aluminum alloy. 2A12-T4 aluminum alloy belongs to the 2XXX series aluminum alloys. It is characterized by high toughness, high strength, low density and good ductility, making it one of the most widely used duralumin alloys in the aerospace field [15]. In contrast, 7A04-T6 aluminum alloy is a 7XXX series ultra-high strength aluminum alloy, which is lightweight and low in density but has relatively poor toughness [16,17]. Aircraft panel structures undertake the functions of load bearing and force transmission. A simple thin-walled skin structure is prone to yielding or buckling under external loads [18]. The aircraft skin is a smooth large-curved component. The 2XXX series aluminum alloys feature good ductility and are easy to process and form, while the 7XXX series aluminum alloys tend to crack during machining due to material brittleness. For the skin structure with longitudinally riveted stiffeners, materials with high stiffness and small deformation are needed to suppress skin failure. The high stiffness of 7XXX series aluminum alloys enables the stiffeners to carry the main load of the panel and reduce the load borne by the skin. Meanwhile, the low toughness of 7XXX series aluminum alloys restricts the skin displacement and reduces the risk of skin failure. 2A10-T4 aluminum alloy rivets are lightweight and high in strength and are commonly used as rivet materials in aviation.

**Figure 1:**
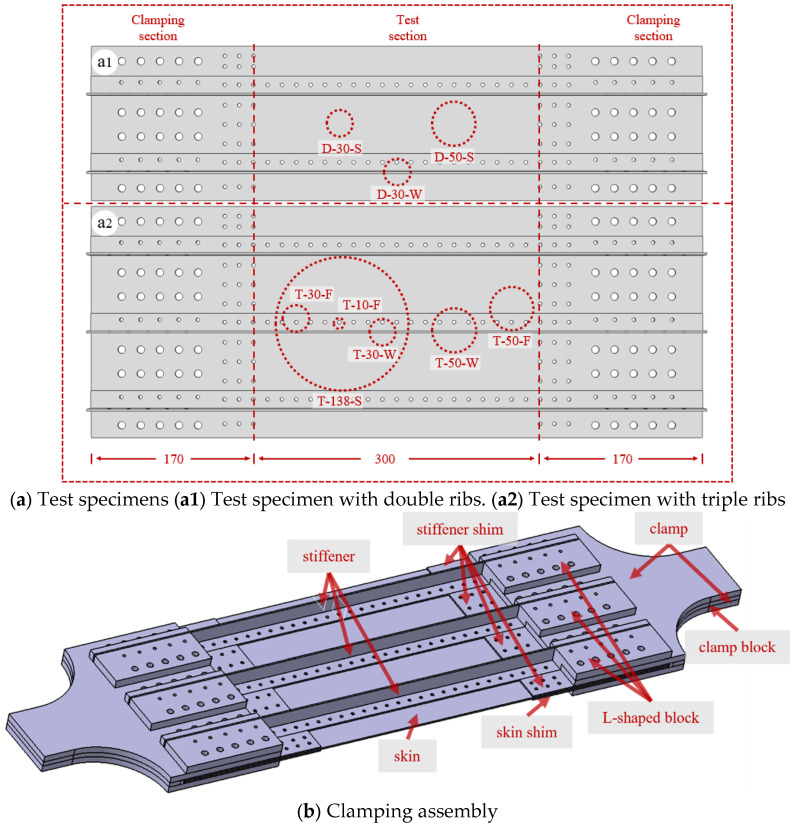
Test specimens and clamp.

The overall length of the specimen panel is 640 mm, with a thickness of 2 mm. The widths of the double-stiffened panel and triple-stiffened panel are 160 mm and 240 mm, respectively. The L-shaped stiffener has a length of 640 mm and a thickness of 2 mm, with a 20 mm width for both the flange plate and the web plate. The skin and stiffeners are riveted together by 4 mm diameter rivets. Two penetration hole diameters (30 mm and 50 mm) are set for the double-stiffened panel, while four diameters (10 mm, 30 mm, 50 mm and 138 mm) are designed for the triple-stiffened panel. Specifically, the penetration hole in the skin damage mode is located at the center of the skin; the hole in the web damage mode penetrates the stiffener web; and the hole in the flange damage mode is located on the flange plate and tangent to the web. All through-holes of the specimens are fabricated using the helical milling process with a CNC milling machine. This process can effectively reduce the residual stress and work hardening degree around the hole edges, minimize the influence of machining technology on the residual strength of the panel, and meanwhile ensure high aperture accuracy, good roundness and nearly burr-free hole edges [19]. The central 300 mm region of the whole specimen is taken as the test section to investigate the panel damage modes under different penetration hole conditions. The 170 mm regions on both sides serve as the clamping sections, in which rows of rivet holes are designed for bolt connection with fixtures. Two bolt hole diameters are adopted, namely 4 mm (small) and 8 mm (large). Speckles are sprayed on the back surface of the specimen panel, and a 2D Digital Image Correlation (DIC) strain measurement system is used to capture the strain nephograms. Figure 1a shows the structure and damage modes of the specimen. Table 1 lists the specimen numbers, where D (dual) and T (triple) represent the number of stiffeners, the number indicates the penetration hole diameter, and S (skin)/W (web)/F (flange) denotes the damage mode.

Figure 1b shows the fixture assembly scheme. The clamp has a width of 100 mm and transitions to the panel width via a rounded corner. The upper and lower clamps grip the two sides of the specimen. Clamp blocks are arranged in the middle to facilitate specimen clamping. Small stiffener shims are inserted between adjacent stiffeners, and large skin shims are used to clamp the front surface of the skin. L-shaped blocks are adopted between the stiffeners and the clamps to connect them so that tensile loads can be applied to both the skin and the stiffeners simultaneously.

**Figure 2:**
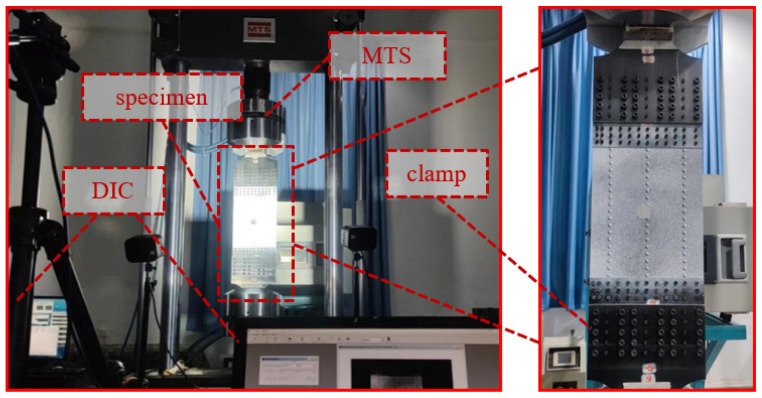
Experimental setup.

The testing apparatus is divided into three modules, including the loading module, the imaging module, and the illumination module. The loading module serves as the core component of the entire experiment and is designed for conducting quasi-static tensile fracture tests on the specimens. This module comprises an MTS-K810 fatigue testing machine (MTS Systems Corporation, Eden Prairie, MN 55344, USA), a cooling chamber, and an oil source pump. Hydraulic grips hold the specimen and apply a uniform tensile load at a rate of 1 mm/min until fracture failure occurs. The testing machine records the load–displacement curve during the tensile process. The imaging module is used to record the tensile fracture failure process of the specimen and capture the variation in the full-field strain nephogram throughout the loading process. This module includes a camera set to capture images at a frequency of 500 ms per frame. The illumination module adjusts the light intensity for the camera to prevent blurred images due to insufficient lighting or overexposure caused by excessive brightness. This module consists of a power supply and two lighting lamps (see Figure 2). The software Vic-2D 7.2.0 is used for subsequent strain nephogram calculation based on the speckle images, allowing observation of the strain variation trends in the specimen during the quasi-static tensile failure process.

## 3. Numerical Simulation Setup

### 3.1. Material Constitutive

The ductile damage criterion [20,21] serves as a phenomenological model describing ductile fracture in metals, effectively simulating the nucleation, growth, and coalescence of voids during tensile deformation. This criterion consists of two distinct phases: ductile damage initiation and ductile damage evolution.

The ductile damage initiation characterizes the onset of material damage, which is typically governed by three key variables: equivalent plastic strain, stress triaxiality, and strain rate.

(1) $${\bar{\varepsilon }}_{D}^{pl}\left(\eta ,{\dot{\bar{\varepsilon }}}^{pl}\right)$$

The stress triaxiality (η) is defined as η=−p/q, where *p* is the pressure stress, *q* is the Mises equivalent stress and ${\bar{\varepsilon }}^{pl}$ is the equivalent plastic strain rate.

State variables are defined to characterize the damage initiation criterion. Ductile damage in metallic materials is considered to initiate when $\omega_{D}=1$ is satisfied.

(2) $$\omega_{D}=\int \frac{d{\bar{\varepsilon }}^{pl}}{{\bar{\varepsilon }}_{D}^{pl}\left(\eta ,{\dot{\bar{\varepsilon }}}^{pl}\right)}=1$$

In finite element analysis, the computational method for each analysis step increment is as follows.

(3) $$\Delta \omega_{D}=\frac{\Delta {\bar{\varepsilon }}^{pl}}{{\bar{\varepsilon }}_{D}^{pl}\left(\eta ,{\dot{\bar{\varepsilon }}}^{pl}\right)}\geq 0$$

The ductile damage evolution characterizes the progressive stiffness degradation of metallic materials, which can be defined as either linear or exponential. The degradation rate can be quantified using either fracture energy or displacement at failure.

The overall damage variable *D* quantifies the cumulative damage in the structural element, where *D* = 1 indicates element failure and removal. The failure process is characterized by both fracture energy and critical displacement, with their relationship established in [22].

(4) $$G_{f}=\int_{{\bar{\varepsilon }}_{oi}}^{{\bar{\varepsilon }}_{f}^{pl}}L\sigma_{y}d{\bar{\varepsilon }}^{pl}$$

**Figure 3:**
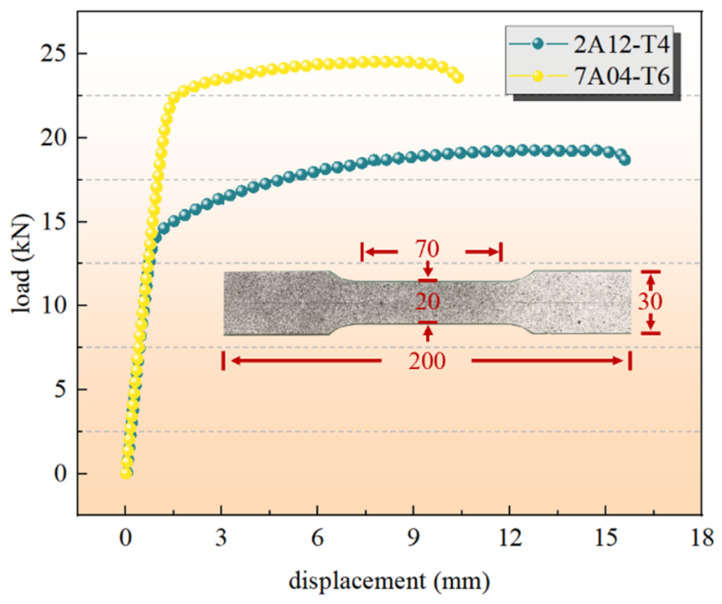
Ductile damage initiation and ductile damage evolution.

The material parameters for 2A12-T4 and 7A04-T6 aluminum alloys are specified in Table 2 and Figure 3. The specimen has a total length of 200 mm, with a clamping section width of 30 mm, a test section width of 20 mm, and a thickness of 2 mm.

### 3.2. Model Setup

**Figure 4:**
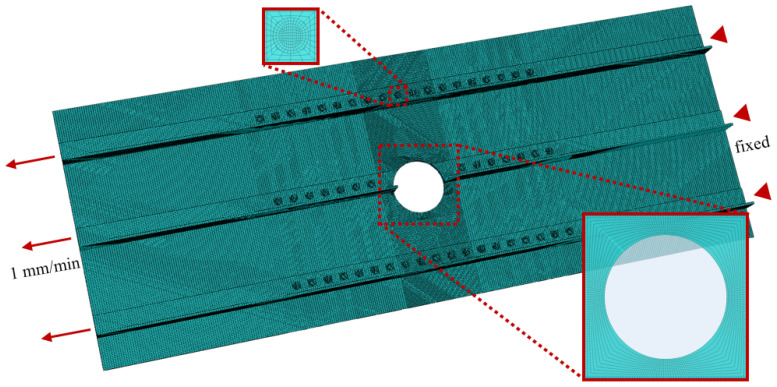
Finite element model setup. The arrow indicates the direction of quasi-static tension at a rate of 1 mm/min.

Finite element modeling and simulation of the stiffened panel test section containing prefabricated perforations were conducted using the commercial software Abaqus 2024. The model dimensions were 640 mm × 240 mm. The global model employed C3D8R elements, while the central test section utilized 1.00 m × 1.00 mm × 1.00 mm, and the two end sections adopted 2.00 m × 1.00 mm × 1.00 mm to balance computational accuracy and efficiency. The mesh around circular holes and rivet holes was refined and uniformly distributed, with element sizes gradually transitioning from 0.6 mm to 1 mm near the perforations. The total number of elements in the finite element model varies slightly under different working conditions—at approximately 329,000. The model is illustrated in Figure 4.

For the riveted connection between the panel and stiffeners, solid rivet models are established only within the test section. A general contact interaction is defined between the panel and stiffeners, with a tangential penalty friction coefficient of 0.2 and normal behavior set as hard contact. Two reference points are created on both sides of the panel and stiffener assembly. The clamping sections on both sides are coupled to their corresponding reference points using kinematic coupling constraints. One end is fully fixed, while the other end is subjected to a tensile displacement load for a specified stretching duration. The tensile rate is set to 1 mm/min to simulate quasi-static conditions. At the tensile end, the displacement, reaction force, and energy of the reference point are output for analysis. This model is solved using the Abaqus/Explicit solver with an explicit dynamic analysis step, which more accurately captures the failure modes and load–displacement response under large-strain conditions.

### 3.3. Model Validation

#### 3.3.1. Mesh Independence Validation

**Figure 5:**
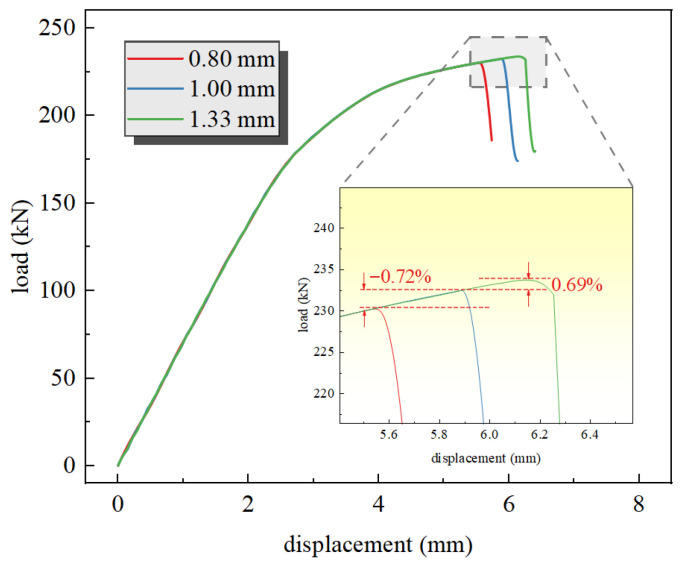
Mesh convergence test.

A mesh sensitivity analysis was conducted on the simulation model S-50-W. The working condition with a hole radius of 5 mm and an eccentricity of 40 mm (where the perforation is located at the center of the stiffened panel) was selected. Three mesh element sizes—0.08 mm × 0.08 mm × 1.00 mm, 1.00 mm × 1.00 mm × 1.00 mm and 1.33 mm × 1.33 mm × 1.00 mm—were applied around the circular hole for simulation calculations. The resulting load–displacement curves from the simulations are shown in Figure 5.

The load–displacement curves for the three mesh sizes exhibit identical variation trends, with the curves completely overlapping prior to failure. As the mesh size decreases, the fracture load remains nearly unchanged, while the fracture displacement shows a minor reduction. The failure displacement setting is related to the mesh size. Without altering the material properties, changes were made to the mesh element failure criterion, resulting in differences in element failure. The errors in fracture load for the 0.80 mm and 1.33 mm meshes compared to the 1.00 mm mesh are −0.72% and 0.69%, respectively, which are nearly negligible. Therefore, the model demonstrates good mesh independence. A mesh size of 1.00 m × 1.00 mm × 1.00 mm was selected to ensure computational accuracy while maintaining computational efficiency.

#### 3.3.2. Experiment and Simulation Comparison

**Figure 6:**
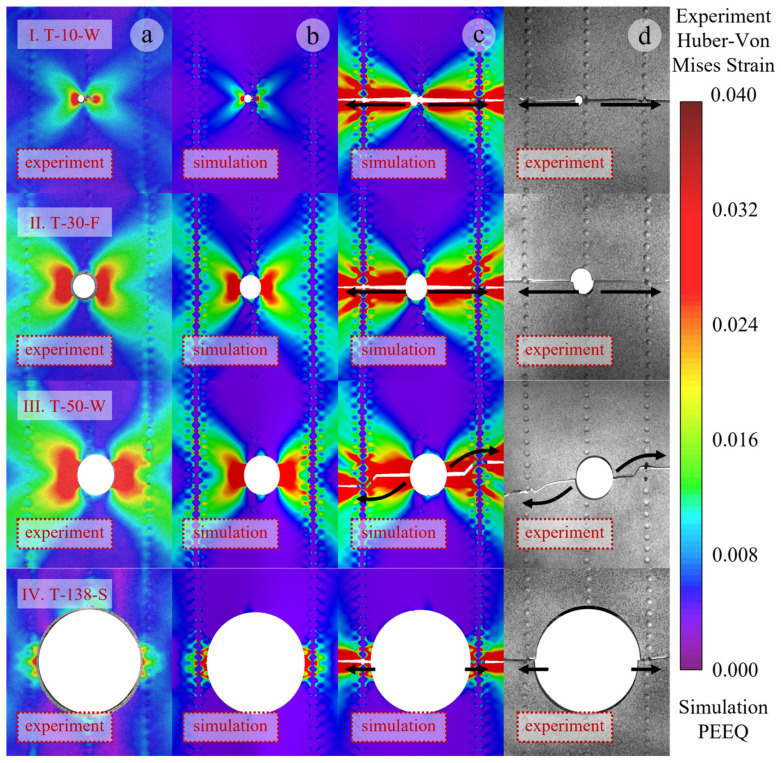
Comparison of partial failure modes. (**a**) Strain cloud diagram of the specimen before fracture. (**b**) Strain cloud diagram of the simulation before fracture. (**c**) Strain cloud diagram of the simulation after complete fracture. (**d**) Complete fracture of specimen. (**I**) Web damaged specimen with a hole diameter of 10 mm. (**II**) Flange damaged specimen with a hole diameter of 30 mm. (**III**) Web damaged specimen with a hole diameter of 50 mm. (**IV**) Skin damaged specimen with a hole diameter of 138 mm.

By observing the strain cloud diagrams and recording the structural fracture sequence, it was found that the 2XXX-series aluminum alloy exhibits better toughness than the 7XXX-series aluminum alloy. Under the same tensile displacement, the stiffeners reach the failure strain first and fracture. Overall, the stiffened panel fails in a sequence where the damaged stiffener fractures first, followed by the skin on both sides of the rupture hole, while the failure sequence of the remaining intact stiffeners and skin remains uncertain. When typical components are observed for changes in the strain cloud diagram, the illustrated cases show that the panels initially fracture at the central damaged stiffener. Subsequently, fractures initiate from both sides of the rupture hole and rapidly propagate toward the lateral stiffeners. The propagating fracture path couples with the rivet holes above and below the center, leading to an oblique fracture. Due to various disturbances, whether the fracture extends to the upper or lower rivet hole is uncertain. In some components, the fracture remains horizontal due to distinct coupling effects between the rupture hole and the rivet holes. The stiffeners exhibit either horizontal or diagonal fracture modes under the influence of the rupture hole. The simulation model accurately predicts the first and second fracture modes of the panel, primarily occurring at the stiffener fractures and the hole-edge fractures, which correspond to the peak load-bearing capacity of the panel structure. However, subsequent failure processes are influenced by multiple factors, such as prior damage, leading to partial discrepancies between simulation and experimental results. Nonetheless, the model effectively captures the dominant failure modes of the panel structure. As shown in Figure 6, the strain distribution before hole-edge failure and the failure modes are compared for selected test specimens. The strain cloud diagrams and failure modes are generally consistent between experiments and simulations. Figure 6a,d display the Huber-Von Mises strain cloud diagram before hole-edge failure and the complete fracture mode of the test specimen, while Figure 6b,c show the plastic strain cloud diagram before hole-edge failure and the corresponding fracture mode under simulation conditions.

The strain nephograms in Figure 6 are analyzed as follows. Under tensile loading, stress concentration occurs at the hole edge of the specimen, which is reflected in the strain nephogram as the maximum strain on both sides of the hole. According to the mechanism analysis in Section 4.1, the maximum strain appears around the ±45° shear bands formed near the hole, showing an X-shaped distribution in the strain nephogram. As the load further increases, the stress around the hole first reaches the yield stress. The material around the hole experiences reduced stiffness due to yielding, and the non-yielded material adjacent to the yielded region enters plastic yielding as the applied load increases. This process propagates outward, and the plastic yielding zone extends from the hole cross-section perpendicular to the tensile direction toward the panel edges. In the strain nephogram, the large strain region expands from the hole edge to both sides of the panel. When the stress at the hole edge reaches the ultimate stress, the structure around the hole begins to undergo rapid failure and fracture along the high-stress region (plastic yielding zone). By analyzing the strain nephograms under different working conditions, two types of structural fracture paths are observed: fracture along the hole cross-section perpendicular to the tensile direction or inclined fracture passing through the rivet holes. According to the mechanism analysis in Section 4.3, the structural fracture path is determined by the stress level comparison between the inclined shear band from the hole edge to the upper (lower) rivet hole and the plastic yielding zone along the hole cross-section perpendicular to the tensile direction, and the plastic zone with a higher stress level is more prone to failure.

The relationship between the strain nephograms and the hole diameter is further analyzed. Figure 6I,II show the working conditions with hole diameters of 10 mm and 30 mm, respectively. A smaller hole exerts a smaller influence on the surrounding material and corresponds to a smaller high-stress region. The inclined shear bands at the hole edge are far from the upper and lower rivet holes on both sides, making it difficult to form effective stress coupling. During the failure and fracture process at the hole edge, the fracture tip propagates rapidly along both sides of the hole cross-section perpendicular to the tensile direction, resulting in a horizontal fracture mode. Figure 6III shows the working condition with a hole diameter of 50 mm. The high-stress region induced by the relatively large hole is close to the upper and lower rivet holes on both sides, forming effective stress field coupling with a higher stress level, which leads to an inclined failure mode passing through the rivet holes. Figure 6IV shows the working condition with a hole diameter of 138 mm. The large hole directly spans between two stiffeners, and the distance from the hole edge to the horizontal rivet holes is smaller than that to the upper and lower rivet holes. A high-stress concentration zone is formed between the hole edge and the horizontal rivet holes, which acts as a weak structural zone. Fracture occurs preferentially at the weak zone, resulting in a horizontal fracture mode.

Some relatively special working conditions exhibit different fracture modes. Specimen T-10-W underwent tensile fracture with a small perforation. Because the perforated cross-section still retains a considerable amount of material, the uneven stress distribution across the entire section is intensified, leading to more stages of sectional fracture failure. The crack progressively propagates from both sides of the perforation until it extends across the entire section. The coupling between the perforation and the rivet hole reduces the overall load-bearing capacity of the panel, resulting in the abnormal phenomenon of a small perforation but weak bearing capacity. Specimen T-138-S features a large perforation spanning the left and right stiffeners. The cross-section where the perforation is located is small, and the stress distribution on this section is more uniform. The asymmetry of the stiffeners causes the entire section on the side of the perforation closer to the web to fracture and fail first, followed by the overall failure of the section on the side farther from the web.

**Figure 7:**
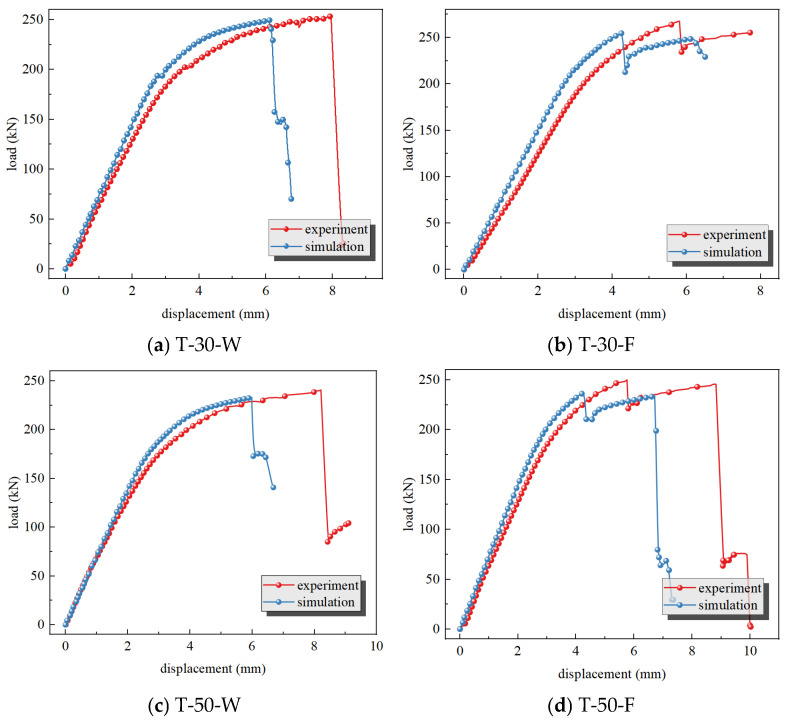
Load–displacement curves and fracture loads comparison.

A comparison of the load–displacement curves between some tests and simulations is shown in Figure 7. Both the experimental and simulated load–displacement curves generally exhibit a trend of initial increase followed by a decrease. In the elastic stage, the load increases rapidly; upon entering the plastic stage, the load increase slows down; finally, upon failure and fracture, the load drops rapidly. This correctly reflects the instantaneous load drop caused by multiple major failure damages in the structure. However, the failure displacement in the simulation is obviously smaller than that in the test, and the reasons are as follows. Under tensile loading, panels with large penetration holes show obvious concave deformation around the holes due to the Poisson effect [23], which increases the additional in-plane shear stress and leads to earlier fracture in the simulation results compared with the test results, while in the actual test this phenomenon is weakened because the fixture constraint restricts the in-plane deformation of the panel. Secondly, the clamping section of the specimen is constrained by the fixtures, and its tensile deformation is basically consistent with that of the fixtures, whereas the influence of the fixtures is ignored in the simulated clamping section, so the tensile displacement of the model is only related to its own material. Finally, the metal around the hole enters the yielding stage first due to stress concentration, and the surrounding material helps the yielded material bear the load; the plastic material propagates from the hole edge along the section perpendicular to the tensile direction until the stress of the whole section reaches the tensile strength, at which point it fractures. The section fracture under test conditions is an integral process, while the simulation model is composed of many finite elements, and the elements at the hole edge are deleted after meeting the failure criterion, which introduces defects into the finite element model, further intensifies the stress concentration and accelerates the failure of the whole finite element model along the section perpendicular to the tensile direction. The error between simulation and test is calculated by Equation (5), where *P*_simulation_ is the simulated failure load and *P*_experiment_ is the experimental failure load. The calculated maximum error of failure load between simulation and test is −5.10%, which accurately reflects the residual strength of the overall structure. Since the main purpose of this simulation is to determine the structural residual strength, the failure displacement does not affect the simulation conclusions.

(5) $$error=\frac{P_{\mathrm{simulation}}-P_{\mathrm{experiment}}}{P_{\mathrm{experiment}}}\times 100\%$$

## 4. Force Interaction and Failure Mechanisms

### 4.1. In-Plane Failure Mechanisms

In an intact panel, the force flow transmits uniformly within the plane (e.g., within the skin plane or the stiffener flange plane). However, a perforation disrupts the structural continuity, leading to an abrupt change in load transmission. The force flow is consequently compelled to transmit along the skin ligament around the hole, resulting in increased shear stress at the hole edge, which exacerbates yielding and failure in this region. The in-plane failure mechanisms of the panel primarily include two types.

**Figure 8:**
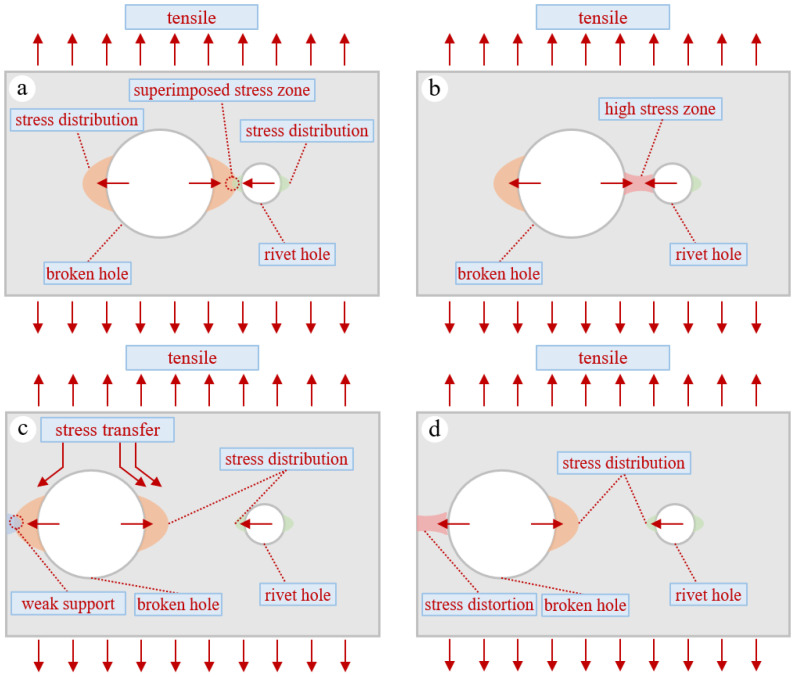
In-plane failure mechanisms. (**a**,**b**) The superposition of stress fields from two holes results in the formation of a high-stress zone. (**c**,**d**) The edge effect causes distortion in the stress field around the hole. The arrows indicate the direction of stress propagation, and the colors contours represent the stress field.

Inter-hole stress concentration effect: When the spacing between two holes is excessively small, the stress concentration zones around their edges overlap and couple with each other, leading to a significant elevation of stress levels in the inter-hole region and the formation of a high-stress band. This markedly increases the stress concentration factor at the cross-section of the circular holes [24]. High stress concentration exacerbates local failure between the two holes under static loading, causing the initial fracture to occur during the static tensile process. For instance, in specimen T-10-W, the close coupling between a perforation on the stiffener flange and a rivet hole induced rapid panel fracture, thereby compromising the overall load-bearing capacity. See Figure 8a,b.

Edge effect: The thin plate edge is a free boundary. The presence of a perforation disrupts the original continuity of the plate edge, causing the load path to sharply redirect around the hole. This results in a redistribution of stress lines that cannot transmit beyond the edge and instead concentrate inward. The material near the hole cannot obtain uniform support from all sides as it would with an internal hole, leading to asymmetric constraints and exacerbated stress distortion on the weaker side of the panel. The stress concentration factor at the circular hole cross-section significantly increases. The outer side of the panel becomes the weak link and fractures first under static tensile loading. See Figure 8c,d. The quasi-static tensile fracture process of a panel containing both a perforation and rivet holes is the result of the coupling of the two effects described above, which is related to the size and location of the perforation. When the perforation is close to a rivet hole, the inter-hole stress concentration effect intensifies. Under static tensile loading, the area between the perforation and the rivet hole fractures first, reducing the load-bearing capacity of the panel. When the perforation is close to the panel edge, the area between the perforation and the panel edge fractures first under static tensile loading, also reducing the panel’s load-bearing capacity. The fracture location affects the load distribution within the remaining structure of the panel, further influencing the failure mode of the panel. For example, in specimen D-30-W, a large eccentricity caused rapid fracture at the panel’s side edge. The support conditions on either side of an aircraft panel may differ, leading to asymmetric stress distribution on the left and right sides. The edge effect is more applicable to support configurations where the constraints on both sides are approximately symmetrical.

**Figure 9:**
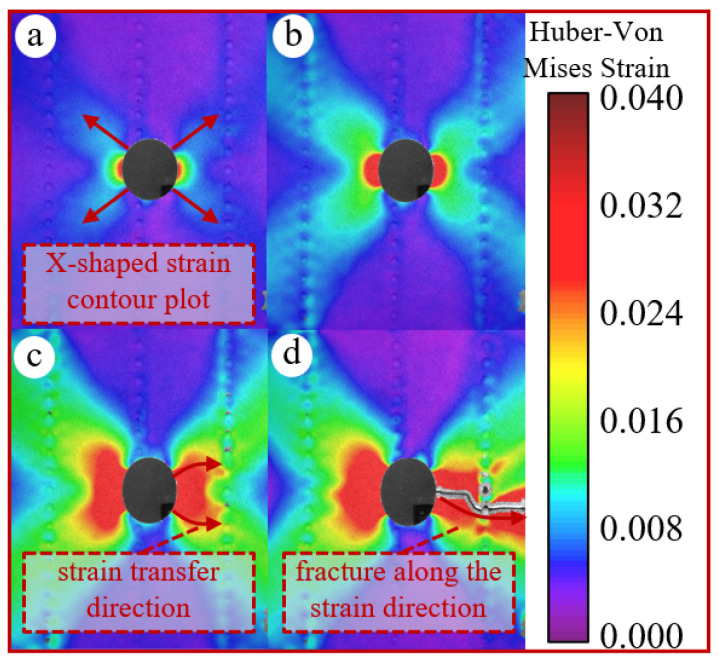
Strain transfer path. (**a**) X-shaped strain nephogram around the hole. (**b**) The X-shaped strain nephogram expands further. (**c**) The strain propagates toward the rivet hole. (**d**) Fracture occurs along the direction of large strain.

During the tensile process, the strain nephogram around the hole exhibits an X-shaped distribution, as shown in Figure 9. Under uniform tensile loading, the circular hole edge experiences a significant stress concentration effect, the tangential stress in the material at the hole periphery first reaches the yield strength, causing it to enter a plastic state and form a local plastic zone. As the external load further increases, the plastic zone expands along the directions of maximum shear stress (oriented at 45° relative to the tensile axis). For perforated plates, the directions of maximum shear stress are typically at ±45° angles to the tensile axis. Plastic deformation preferentially occurs along these two diagonal directions, forming symmetrical plastic slip bands. These bands manifest as high-strain regions in the strain field, resulting in the characteristic X-shaped distribution observed in the strain nephogram. This mechanism also explains why perforated panels may undergo fracture propagation along inclined directions.

### 4.2. Interaction Mechanism Between Skin and Stiffener

**Figure 10:**
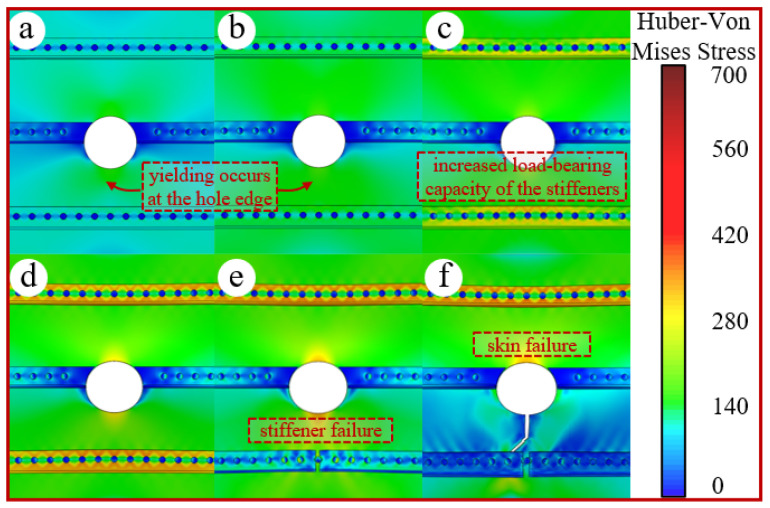
Stress variation process. (**a**) Yielding initiates around the hole. (**b**) Further yielding occurs around the hole. (**c**) The load bearing capacity of the stiffeners increases. (**d**) The load carried by the stiffeners further increases. (**e**) The stiffeners fail first. (**f**) The skin fails subsequently.

The skin and the stiffener essentially form an axial tension-shear coupling parallel system. During the elastic tensile stage, the external load is distributed between them according to their respective stiffnesses [25], as given by the distribution Formula (6). Owing to the skin’s large cross-sectional area, the external load in the elastic stage is primarily borne by the skin portion (see Figure 10a,b). Here, *P* denotes the external load, *E* is the material’s elastic modulus, and *A* represents the material’s cross-sectional area, with the subscripts *skin* and *stiff* indicating the panel’s skin and stiffener, respectively.

(6) $$\left\{\begin{array}{l} P_{\mathrm{skin}}+P_{\mathrm{stiff}}=P\\ \frac{P_{\mathrm{skin}}}{P_{\mathrm{stiff}}}=\frac{E_{\mathrm{skin}}A_{\mathrm{skin}}}{E_{\mathrm{stiff}}A_{\mathrm{stiff}}} \end{array}\right.$$

Due to its relatively lower yield strength, the skin enters the plastic stage first, leading to a decline in its stiffness and a gradual reduction in its load-bearing capacity. The load is subsequently transferred to the stiffeners through rivet shear forces and interfacial friction, causing the stiffeners to progressively dominate in carrying the external load. Furthermore, the external load becomes concentrated on the stiffener webs, which possess higher stiffness, as shown in Figure 10c,d. Once the stress in the stiffener section reaches the ultimate stress, it undergoes instantaneous failure and fracture. The abrupt unloading of the stiffener transfers load to the skin via rivet shear forces, as shown in Figure 10e,f. The instantaneous impulse imposed on the skin may lead to its sudden failure, which macroscopically manifests as the simultaneous rupture of both the stiffener and the skin.

**Figure 11:**
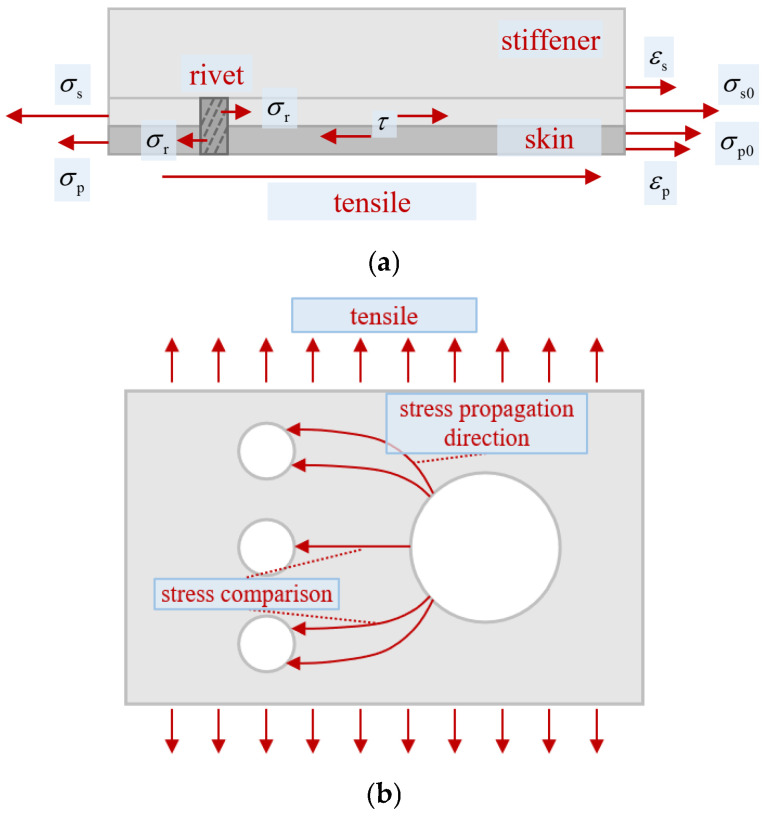
Interaction mechanism. (**a**) Interaction between skin and stiffener [10]. (**b**) Interaction between broken hole and rivet hole.

The stress analysis between the skin and stiffener is shown in Figure 11a. The 2XXX-series aluminum alloy has a lower yield strength but higher fracture toughness compared to the 7XXX-series aluminum alloy. Under external loading, the strain in the skin of the stiffened panel exceeds that in the stiffener, resulting in relative displacement between them. The interfacial friction τ and the normal compressive stress *σ*_r_ from rivet squeezing counteract this displacement, maintaining the overall quasi-static equilibrium of the stiffened panel, which satisfies the equilibrium Formula (7). Here, *σ*_s_ denotes the axial tensile stress in the stiffener near the central side of the panel, *σ*_p_ represents the axial tensile stress in the skin near the central side, *σ*_s0_ indicates the axial tensile stress in the stiffener near the boundary side of the panel, and *σ*_p0_ signifies the axial tensile stress in the skin near the boundary side.

(7) $$\left\{\begin{array}{l} \sigma_{s}=\sigma_{r}+\tau +\sigma_{s0}\\ \sigma_{p}+\sigma_{r}+\tau =\sigma_{p0} \end{array}\right.$$

According to Formula (7), from the panel boundary to the panel center, the axial tensile stress of the stiffener gradually increases, while the axial tensile stress of the skin gradually decreases. Consequently, the stiffener tends to fracture first near the central region [10].

For the rivets between the skin and stiffeners, the rivets bear relatively small forces during the elastic stage. The preload provided by the pull-type rivets compacting the panels primarily increases the friction between the skin and stiffeners. The external load is initially resisted by the inter-plate friction against the separation of the skin and stiffeners. When the external load exceeds the maximum static friction between the panels, the shear stress overcoming panel separation is gradually compensated by the normal stress from the rivets compressing the inner wall of the hole.

During the initial load-bearing stage of the rivets, the rivets at both loading ends experience the highest forces. Subsequently, plastic deformation around the hole allows for local slip, leading to a more uniform distribution of rivet shear forces toward the center of the panel. During the plastic stage, the peak shear force around the hole decreases compared to the elastic stage, but the overall shear force value increases. However, the yield stiffness around the hole declines, causing the load to gradually transfer to the stiffeners, ultimately resulting in stiffener fracture.

### 4.3. Fracture Propagation Direction Mechanisms in Perforated Panels

The interaction between the perforation on the skin and the bilateral rivet holes influences the fracture propagation direction of the skin. The fracture may propagate and rupture at a certain inclination angle, as shown in Figure 9. During the tensile process, the area around the hole first enters the plastic state, exhibiting a plastically expanded zone extending in the X-direction. The coupling of stiffener shear and far-field tensile stresses forms a combined tension-shear stress state around the hole periphery. This influences the initiation of plastic expansion, which occurs at approximately 15–45° around the hole (measured from the axis perpendicular to the tensile direction). The plastic expansion zone may preferentially couple adjacently with the upper rivet hole, forming a high-stress band directly between the two holes. This induces microcrack formation between the holes, leading to an inclined fracture surface. On the other hand, after the formation of the upper and lower plastic zones around the perforation, the central region of the hole progressively enters the plastic stage and expands in the direction perpendicular to the tensile load. This gradually forms a high-stress zone between the central interface of the perforation and the central rivet hole, inducing microcrack formation between the holes and resulting in a horizontal fracture surface. The dominant modes of these two high-stress bands manifest as two distinct fracture patterns. The geometric structure of the panel or any initial defects (e.g., scratches, microcracks around rivet holes) can influence the dominance of these two stress bands. The mechanism is illustrated in Figure 11b.

**Figure 12:**
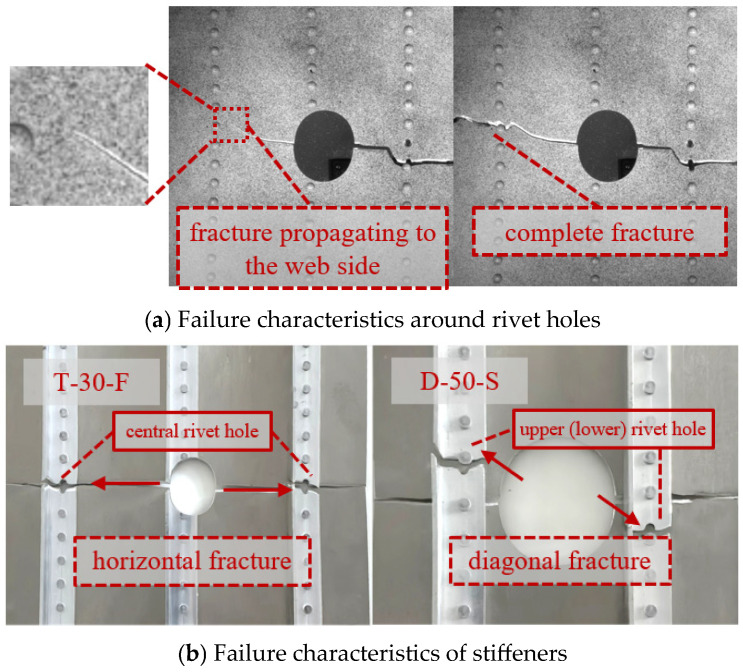
Failure characteristics.

During the fracture process, a phenomenon occurs where the fracture surface first propagates to the vicinity of the contact surface between the rivet hole periphery and the stiffener, and then fractures along the cross-section. This is particularly evident when the web is located on the central side of the panel and in close proximity to the perforation, as shown in Figure 12. Under tensile loading, the displacement of the stiffener is less than that of the skin (as the 7000-series aluminum alloy is more brittle than the 2000-series aluminum alloy). The skin generates a shear stress opposite to the tensile direction, which weakens the effect of the tensile load and inhibits structural failure and damage. Due to the high stiffness of the web plate, which carries more load, the displacement difference at the contact surface increases, leading to a rise in shear stress and making the inhibitory effect more pronounced.

Based on the analysis of the interaction between the skin and the stiffener, under normal circumstances, the stiffener should fracture first at the rivet hole located in the central region of the stiffener. However, some stiffeners exhibit a diagonal fracture mode, as shown in Figure 12b.

The stress distribution in the stiffener is altered by the changed stress distribution in the perforated skin. Under three-dimensional conditions, this is influenced by both the stresses perpendicular to the flange direction within the web and the interfacial stresses between the stiffener and the skin. The stiffness disparity between the skin and stiffener first induces stress concentration at their contact interface. Because the perforated skin exhibits an X-shaped stress field distribution, the upper (lower) rivet hole of the skin experiences higher axial skin stress. This increases the stress gradient between the upper (lower) rivet hole and the central rivet hole. The heightened shear stress between the stiffener and skin under these elevated stress conditions leads to an increase in axial stress at the location of the upper (lower) rivet hole on the stiffener, forming a new stress concentration zone. Consequently, the upper (lower) rivet hole on the stiffener may fracture prior to the central rivet hole.

## 5. Residual Strength and Evaluation Methods for Stiffened Panel Structures

### 5.1. Residual Strength of Stiffened Panels

**Figure 13:**
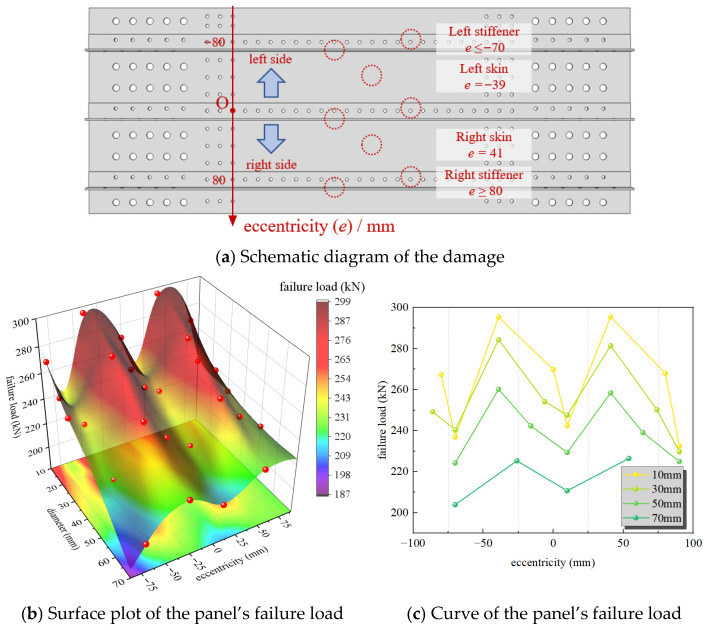
Failure loads comparison. Red dots represent the failure loads under different working conditions.

The simulation calculated a total of 27 working conditions to compare the residual strength of the panel structure under different rupture hole sizes and locations. A comparison of the failure loads for different hole sizes and positions is shown in Figure 13.

Overall, for panels with rupture holes of the same size, the residual strength is lowest for those with stiffener web damage, followed by those with stiffener flange damage, and is highest for those with only skin damage. The web, as the primary high-stiffness component of the stiffener, carries the main load. Additionally, web damage results in the loss of a larger cross-sectional material area around the rupture, severely weakening the overall load-bearing capacity of the panel. For panels with different rupture sizes, the general trend is that larger openings lead to lower residual strength. Large openings reduce the load-transferring “bridge” of skin material, diminish constraint due to material loss, decrease the stiffness for free deformation of the remaining bridge, and reduce load-bearing capacity. This shifts a greater load share to the stiffeners, making them more prone to fracture. However, an anomaly is observed where the residual strength for the 10 mm web damage case is lower than that for the 30 mm hole web damage case. This is because the 10 mm hole is located close to a rivet hole, creating a high-stress band between the holes that induces premature yielding and fracture of the material bridge, leading to an abnormal reduction in failure load. Although the L-shaped stiffener is geometrically asymmetric along the panel’s length, Figure 13c shows that the load variation at symmetric positions is not significant. This indicates that the stiffeners act as the primary load-bearing components, and the overall failure load of the panel is determined by the damage state of these stiffeners, rather than minor geometric asymmetries. A comparison of the failure loads at different failure locations is provided in Figure 13.

Comparing the failure displacements corresponding to the maximum failure loads under the same perforation size, the maximum failure displacement of the panel with stiffener web damage is greater than that of the panel with stiffener flange damage. When the high-stiffness web remains undamaged, the axial constraint provided by the web generates a bridging effect. The stiffener spans the perforated area, inhibiting the axial expansion of the material around the hole, constraining the overall displacement of the panel. The stiffener provides constraint force through the rivet cluster, reducing the displacement around the hole and increasing the load-bearing capacity. A comparison of the maximum failure displacements is shown in Table 3.

### 5.2. Residual Strength Evaluation Methods for Stiffened Panels

For the residual strength assessment of stiffened panels containing smooth circular holes, under certain working conditions during the tensile process, the fracture propagates at an inclined angle. However, prior to the primary fracture of the structure, the stress is predominantly concentrated on the cross-section where the perforation is located. The localized inclined stress bands in other areas do not significantly affect the overall structural failure and fracture. Therefore, the net-section criterion remains valid. Nevertheless, the net-section failure criterion or the net-section yield criterion [8] assumes that failure occurs when both the skin and the stiffener of the panel simultaneously reach the ultimate strength at the weakened cross-section. In actual tensile processes, variations in the panel structure or the location and size of perforations lead to uneven load transfer paths and stress field distributions. Specific areas, such as those discussed in Section 4.1, may experience premature failure and fracture due to stress concentration. Simply applying the net-section criterion can result in errors exceeding 20%, severely compromising the reliability of residual strength assessment. Thus, effective modification of the net-section criterion is essential.

**Figure 14:**
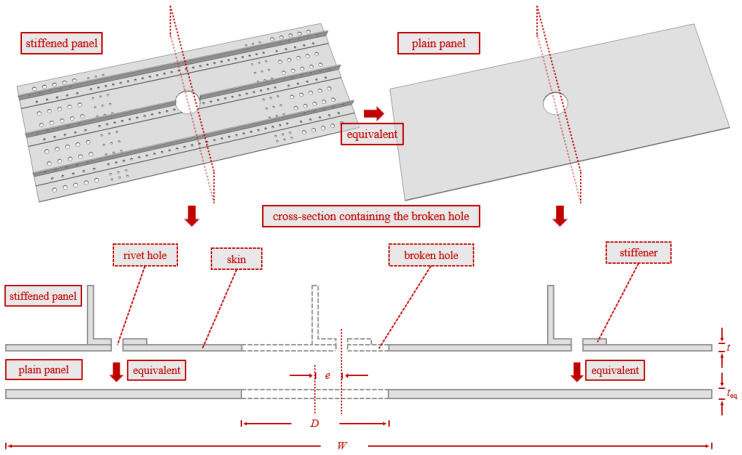
Stiffened panel equivalence. The arrows represent the equivalent process.

The ultimate strength of the material is selected as the criterion for structural fracture. The perforated stiffened panel is equivalently represented as a plain panel of the same size as the skin but with varying thickness, containing a perforation. The location and size of the perforation correspond to those on the skin of the original stiffened panel. The equivalent diagram is shown in Figure 14. The cross-section where the perforation is located is selected. The ultimate strength of the skin and the stiffener is used to equivalently transform the cross-sectional area of the stiffener into that of the skin, as shown in Formula (8). Here, *A*’_skin_ represents the equivalent cross-sectional area of the stiffener transformed into the skin, *A*_stiff_ is the original cross-sectional area of the stiffener, and all cross-sectional areas must exclude the areas of the rivet holes and the perforation. *σ*_tb_ is the ultimate strength of the stiffener, and *σ*_kb_ is the ultimate strength of the skin.

(8) $$A_{\mathrm{skin}}^{\prime }=\frac{A_{\mathrm{stiff}}\sigma_{\mathrm{tb}}}{\sigma_{\mathrm{kb}}}$$

The converted total cross-sectional area of the skin is given by Formula (9), where *A*_skin_ represents the original cross-sectional area of the skin.

(9) $$A_{\mathrm{total}}=A_{\mathrm{skin}}+A_{\mathrm{skin}}^{\prime }$$

The equivalent plain skin thickness is given by Formula (10), where *W* represents the width of the skin panel and *D* denotes the perforation diameter.

(10) $$t_{\mathrm{eq}}=\frac{A_{\mathrm{total}}}{W-D}$$

In engineering, a length ratio of less than 0.1 is generally regarded as a small size, and the condition where the ratio of eccentricity to panel width is less than 0.1 is considered to indicate that the penetration hole is located near the center of the panel. When the ratio of eccentricity to panel width is greater than 0.1, the hole is regarded as having a large eccentricity. For a plain panel, excessive eccentricity of the penetration hole leads to non-uniform stress distribution on both sides of the cross-section where the hole is located, and the weaker side (with a smaller cross-section) reaches the ultimate strength first due to stress concentration. The Stress Averaging Factor α is introduced to characterize the stress level of the stronger side (with a larger cross-section), which is expressed as the stress ratio of the cross-sections on both sides of the hole before structural fracture failure. At this point, Equation (11) is valid before structural fracture, where *e* represents the eccentricity and *P*_f_ denotes the failure load.

(11) $$\sigma_{\mathrm{kb}}\left(\frac{W}{2}-\frac{D}{2}-e\right)t_{\mathrm{eq}}+\alpha \sigma_{\mathrm{kb}}\left(\frac{W}{2}-\frac{D}{2}+e\right)t_{\mathrm{eq}}=P_{f}$$

The residual strength of the structure can be evaluated by means of the given stress averaging coefficient. The equivalent cross-sectional area of the damaged hole is extracted and denoted as Formula (12).

(12) $$A_{\mathrm{eq}}=\left[\left(\frac{W}{2}-\frac{D}{2}-e\right)+\alpha \left(\frac{W}{2}-\frac{D}{2}+e\right)\right]t_{\mathrm{eq}}$$

When the ratio of eccentricity to panel width is less than or equal to 0.1, the hole is regarded as having a small eccentricity, that is, the hole is located at the center of the panel. When the penetration hole is positioned near the center of the panel, the difference in stress distribution on both sides of the hole is reduced, and the direct adoption of the stress averaging factor cannot properly reflect the stress difference around the hole of the panel. Regarding the material around the hole as a whole, a conversion factor *β* is introduced to characterize the strength loss coefficient when a stiffened panel with a central hole is equivalent to a flat plate. The equivalent cross-sectional area of the penetration hole is expressed by Equation (13).

(13) $$A_{\mathrm{eq}}=\beta \left(W-D\right)t_{\mathrm{eq}}$$

Combined with the above discussion on dimensions, the calculation of the equivalent area is summarized in Equation (14).

(14) $$A_{\mathrm{eq}}=\left\{\begin{array}{ll} \beta \left(W-D\right)t_{\mathrm{eq}}, & 0\leq \frac{e}{W}\leq 0.1\\ \left[\left(\frac{W}{2}-\frac{D}{2}-e\right)+\alpha \left(\frac{W}{2}-\frac{D}{2}+e\right)\right]t_{\mathrm{eq}}, & 0.1<\frac{e}{W}<1 \end{array}\right.$$

However, certain structural configurations, such as closely spaced holes or holes near edges, may lead to premature structural failure and fracture due to excessive local stress concentration. When the distance between the hole edge and any boundary—including the boundary between a hole in the skin and the panel skin edge, the boundary between a hole in a stringer and the flange, or the boundary between a hole and other holes such as rivet holes—is considered small, a structural weakening factor *γ* is introduced to correct the reduction in load-bearing capacity caused by strength weakening of vulnerable structures. Similar to the structural strength margin, its value is an engineering empirical value. Combined with engineering experience regarding small sizes, assuming the distance between the boundaries of circular holes is denoted as *l*, a spacing-to-width ratio of less than 0.1 is defined as a small size. The value of *γ* is given by Formula (15).

(15) $$\gamma =\left\{\begin{array}{c} 0.9,\;0<\frac{l}{W}\leq 0.1\\ 1.0,\;0.1<\frac{l}{W}<1 \end{array}\right.$$

With the introduction of the weakening factor *γ*, the failure criterion for the panel structure is given by Formula (16), where *P* represents the external load.

(16) $$\sigma_{\mathrm{eq}}=\frac{P}{A_{\mathrm{eq}}}\leq \sigma_{\mathrm{kb}}$$

The residual strength (failure load) of the panel structure is estimated by Formula (17).

(17) $$P_{\mathrm{evaluatin}}=\sigma_{\mathrm{kb}}A_{\mathrm{eq}}$$

**Figure 15:**
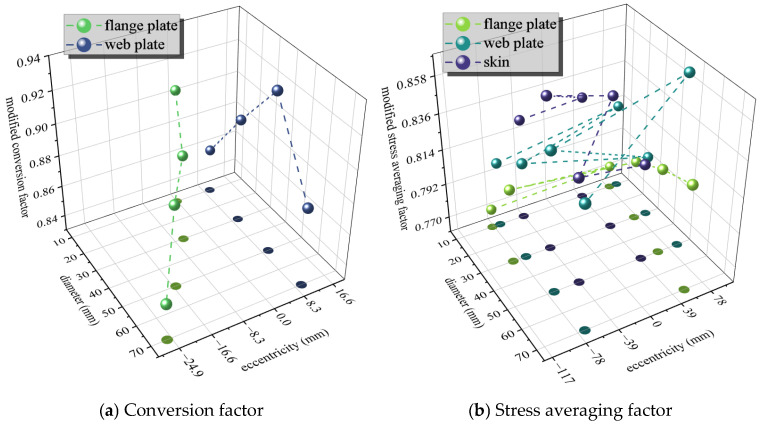
Conversion factor and stress averaging factor.

The residual strength of the 27 working conditions from both experiments and simulations was assessed using the aforementioned method. The stress averaging factor was applied to cases with larger eccentricity, while the conversion factor was used for those with smaller eccentricity. The calculation follows Formula (18), where the structural weakening coefficient *γ* is set to 0.9. The computational results are presented in Figure 15.

(18) $$\left\{\begin{array}{ll} \beta =\frac{P_{f}}{\sigma_{\mathrm{kb}}t_{\mathrm{eq}}\gamma \left(W-D\right)}, & 0\leq \frac{e}{W}\leq 0.1\\ \alpha =\frac{\frac{2P_{f}}{\sigma_{\mathrm{kb}}t_{\mathrm{eq}}\gamma }-W+D+2e}{W-D+2e}, & 0.1<\frac{e}{W}<1 \end{array}\right.$$

The empirical stress averaging factors under flange damage, web damage and skin damage are calculated, respectively. The relatively small values in the concentrated region of the factors under various working conditions are adopted to ensure a conservative estimate while meeting the accuracy requirements (slightly dangerous estimates are allowed). All empirical stress averaging factors in the present evaluation are given in Formula (19). As shown in Figure 15b, the stress averaging factor is generally lower for stringer damage (including flange and web damage) than for skin damage. The stress averaging factor characterizes the difference in stress distribution between the remaining structure and the failed local structure at the point of local failure. A smaller stress averaging factor indicates a more non-uniform distribution. In stiffened panel structures, the stringers serve as the primary load-bearing components and possess a higher load-bearing capacity than the skin. Consequently, when failure occurs at the hole edge, the remaining section with stronger load-bearing capacity exhibits a higher average stress level, resulting in a larger stress averaging factor.

**Figure 16:**
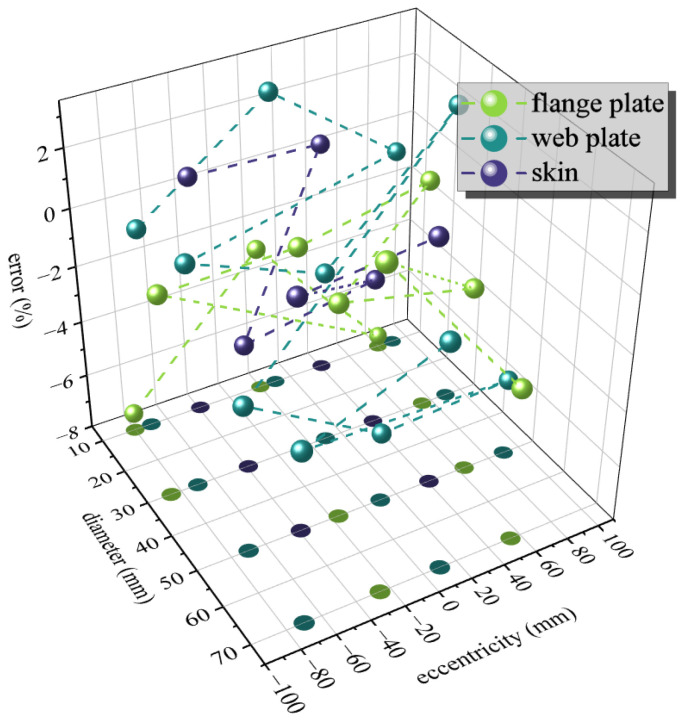
Evaluation error.

Comparing the empirical conversion factors for flange damage and web damage reveals that their overall levels are quite similar. The relatively small values in the approximate concentrated region of all conversion factors are adopted, so as to meet the accuracy requirement and achieve a conservative estimate as much as possible. Thus, we obtain: *β* = 0.88. Combining this with the *γ* value rules, the residual strength assessment errors are calculated and shown in Figure 16. The maximum error is −7.57%, with only five groups resulting in non-conservative estimates. The maximum error among these non-conservative estimates is 2.53%, and overall, the errors are generally controlled within 2%. This indicates that the assessment method provides a conservative estimate on the whole.

(19) $$\alpha =\left\{\begin{array}{ll} 0.80, & \mathrm{flange}\;\mathrm{plate}\\ 0.82, & \mathrm{web}\;\mathrm{plate}\\ 0.83, & \mathrm{skin} \end{array}\right.$$

Metallic structures with holes exhibit similar stress distributions under loading. The core of the stress averaging factor *α* and the conversion factor *β* lies in the stress distribution of the structure before failure. The same evaluation method can be adopted for different metallic materials, although α and *β* may require minor adjustments. The structural weakening coefficient *γ* is an engineering correction parameter for vulnerable structures, similar to the structural strength margin, and no further correction is needed.

A case with a diameter of 70 mm and an eccentricity of 26 mm is selected to verify the rationality of defining a small size (with a length ratio of 0.1). The eccentricity of 26 mm is close to 24 mm (which is 0.1 of the 240 mm panel width). Using the two methods provided in Equation (20), the estimated residual load-bearing capacities of the damaged structure are 229.44 kN and 232.24 kN, respectively, resulting in an error of 1.2% between them. This, to some extent, indicates the rationality of the engineering empirical selection for defining a small size.

(20) $$error=\frac{P_{\mathrm{evaluation}}-P_{\mathrm{experiment}}}{P_{\mathrm{experiment}}}\times 100\%$$

## 6. Conclusions

This study designed three types of double-stiffened panels and six types of triple-stiffened panels containing perforations and conducted quasi-static tensile residual strength tests on typical aircraft aluminum alloy stiffened panels to investigate the load transfer paths during the tensile failure process and the variation patterns of the stress–strain fields under increasing external loads. Using a ductile damage model, finite element simulation software was employed to simulate the tensile fracture of the stiffened panels. The failure modes and residual strength obtained from the simulations showed good agreement with the experimental results. Through both experimental and simulation approaches, the failure mechanisms of the stiffened panel structures were elucidated, and an effective residual strength assessment method was proposed by modifying the net-section failure criterion, achieving an evaluation accuracy within 7.57%. Based on this, the following conclusions were drawn:

1. Under specific structural configurations, premature failure of the panel occurs: the adjacency of stress fields between the perforation and the rivet hole induces a high-stress band between the holes, leading to early failure of the skin ligament; the coupling of an edge-proximal perforation with the panel boundary causes stress concentration at the edge, resulting in reduced load-bearing capacity in the boundary region.
2. During the tensile process, influenced by various disturbances, the fracture may exhibit either horizontal or inclined fracture patterns. The perforation compromises the structural integrity, causing stress to propagate along the maximum shear direction, forming inclined plastic slip bands. The high-stress field exhibits an “X-shaped” distribution, interacting with the central rivet hole and the upper (lower) rivet holes to form two high-stress bands. The band with the higher stress level fails preferentially, resulting in either horizontal or inclined failure modes.
3. The skin and stiffener interact through friction at the contact surface and the normal pressure of rivets. Toward the central direction of the panel, the axial stress of the skin gradually decreases, while the axial stress of the stiffener gradually increases. The central rivet hole of the stiffener is a fracture-sensitive region, typically exhibiting horizontal fracture at the center of the stiffener. However, the perforation on the skin increases the axial stress around it and reduces the stress concentration at the center of the stiffener, potentially leading to a diagonal fracture mode in the stiffener.
4. The overall residual strength of the panel with stiffener web damage is lower than that with stiffener flange damage under the same perforation size. Generally, a larger perforation results in a lower residual strength of the panel; however, certain special structural configurations, such as dual-hole coupling or edge-proximal perforations, can further reduce the residual strength of the panel. Due to the bridging effect of the panel, the failure displacement corresponding to the maximum failure load of the panel with stiffener web damage is less than that of the panel with stiffener flange damage.
5. Based on the net-section failure criterion, the overall accuracy of residual strength assessment for stiffened panels has been improved by introducing the stress averaging factor *α*, the conversion factor *β*, and the structural weakening factor *γ*. The overall assessment error is controlled within 2%, with a maximum individual error of 7.57%.

## Figures and Tables

**Table 1:** Experimental design of perforated aluminum alloy panels.

Specimen Numbering	Stiffeners Number	Perforation Diameter/mm	Eccentricity/mm	Damage Location
D-30-S	2	30	0	skin
D-30-W	2	30	50	web plate
D-50-S	2	50	0	skin
T-10-W	3	10	10	web plate
T-30-W	3	30	10	web plate
T-30-F	3	30	6	flange plate
T-50-W	3	50	10	web plate
T-50-F	3	50	16	flange plate
T-138-S	3	138	0	skin

**Table 2:** A12-T4 and 7A04-T6 aluminum alloy material parameters [10].

Aluminum Alloy	Elastic Modulus	Poisson’s Ratio	Failure Strain	Tensile Strength
2A12-T4	69 GPa	0.33	0.20	481 MPa
7A04-T6	72 GPa	0.33	0.14	614 MPa

**Table 3:** Maximum failure displacement comparison.

Specimen Number	Damage Location	Perforation Diameter/mm	Maximum Failure Displacement/mm
T-30-W	web plate	30	7.95
T-50-W	web plate	50	8.21
T-30-F	flange plate	30	5.81
T-50-F	flange plate	50	5.76

## Data Availability

The original contributions presented in this study are included in the article. Further inquiries can be directed to the corresponding author.
